# Concurrent IgG4-related hypophysitis and clinically nonfunctioning gonadotroph pituitary neuroendocrine tumor

**DOI:** 10.1186/s12902-023-01353-y

**Published:** 2023-05-04

**Authors:** Shigeyuki Tahara, Robert Yoshiyuki Osamura, Yujiro Hattori, Eitaro Ishisaka, Chie Inomoto, Hitoshi Sugihara, Akira Teramoto, Akio Morita

**Affiliations:** 1 Department of Neurological Surgery, Graduate School of Medicine, Nippon Medical School, 1-1-5Bunkyo-Ku, SendagiTokyo, 113-8603 Japan; 2 Department of Pathology, Nippon Koukan Hospital, Kanagawa, Japan; 3 Department of Anatomy and Neurobiology, Graduate School of Medicine, Nippon Medical School, Tokyo, Japan; 4 Department of Pathology, Tokai University School of Medicine, Kanagawa, Japan; 5 Division of Diabetes, Endocrinology and Metabolism, Department of Medicine, Nippon Medical School, Tokyo, Japan; 6 Shonan University of Medical Sciences, Kanagawa, Japan

**Keywords:** Immunoglobulin G4, Hypophysitis, Pituitary neuroendocrine tumor, Case report

## Abstract

**Background:**

Some patients develop immunoglobulin G4 (IgG4)-related hypophysitis associated with systemic diseases. More than 30 cases of IgG4-related hypophysitis have been reported. However, biopsy has rarely been performed in these patients, and none have had an associated pituitary neuroendocrine tumor (PitNET). We present a case of concurrent IgG4-related hypophysitis and PitNET.

**Case presentation:**

A 56-year-old Japanese man arrived at the hospital with visual impairment, bitemporal hemianopia, and right abducens nerve palsy. Magnetic resonance imaging revealed pituitary body and stalk swelling as well as a small poorly enhanced right anterior lobe mass. Laboratory and loading test results suggested hypopituitarism. Because IgG4 level was elevated, a systemic examination was performed; multiple nodules were found in both lung fields. The diagnosis was based on an endoscopic transnasal biopsy of the pituitary gland. A histopathological examination revealed a marked infiltration of plasma cells into the pituitary gland, which was strongly positive for IgG4. The histological features of the resected tumor were consistent with those of gonadotroph PitNET, which was immunohistochemically positive for follicle-stimulating hormone-β and steroidogenic factor-1, and no plasma cell infiltration was observed. Based on the histopathological examination results, steroid therapy was initiated, which reduced pituitary gland size and serum IgG4 levels.

**Discussion and Conclusions:**

This is the first reported case of IgG4-related hypophysitis with PitNET. Although no pathological findings indicating a relationship between the two conditions were found, we were able to preoperatively differentiate multiple lesions via detailed diagnostic imaging.

## Background

Immunoglobulin G4 (IgG4)-related disease is a systemic chronic inflammatory condition characterized by elevated IgG4 levels and a marked infiltration of IgG4-positive plasma cells into affected organs. This disease was first reported in 2001 in a study on autoimmune pancreatitis associated with high serum IgG4 levels [1]. It was later discovered this disease could affect other regions of the body. In the central nervous system, the disease causes hypophysitis, hypertrophic pachymeningitis, and intracranial neoplastic lesions. Hypophysitis was first reported in 2004 [2], and other types of lesions have since been reported in other studies. Although studies have described cases of IgG4-related disease complicated by malignant neoplasms and the relationship between the two, no studies have examined cases complicated by a pituitary neuroendocrine tumor (PitNET), a type of benign neoplasm. Herein, based on pathological findings, we report a case of a patient with IgG4-related hypophysitis complicated by PitNET, which was discovered because of hypopituitarism and a visual function disorder.

## Case presentation

**Fig. 1:**
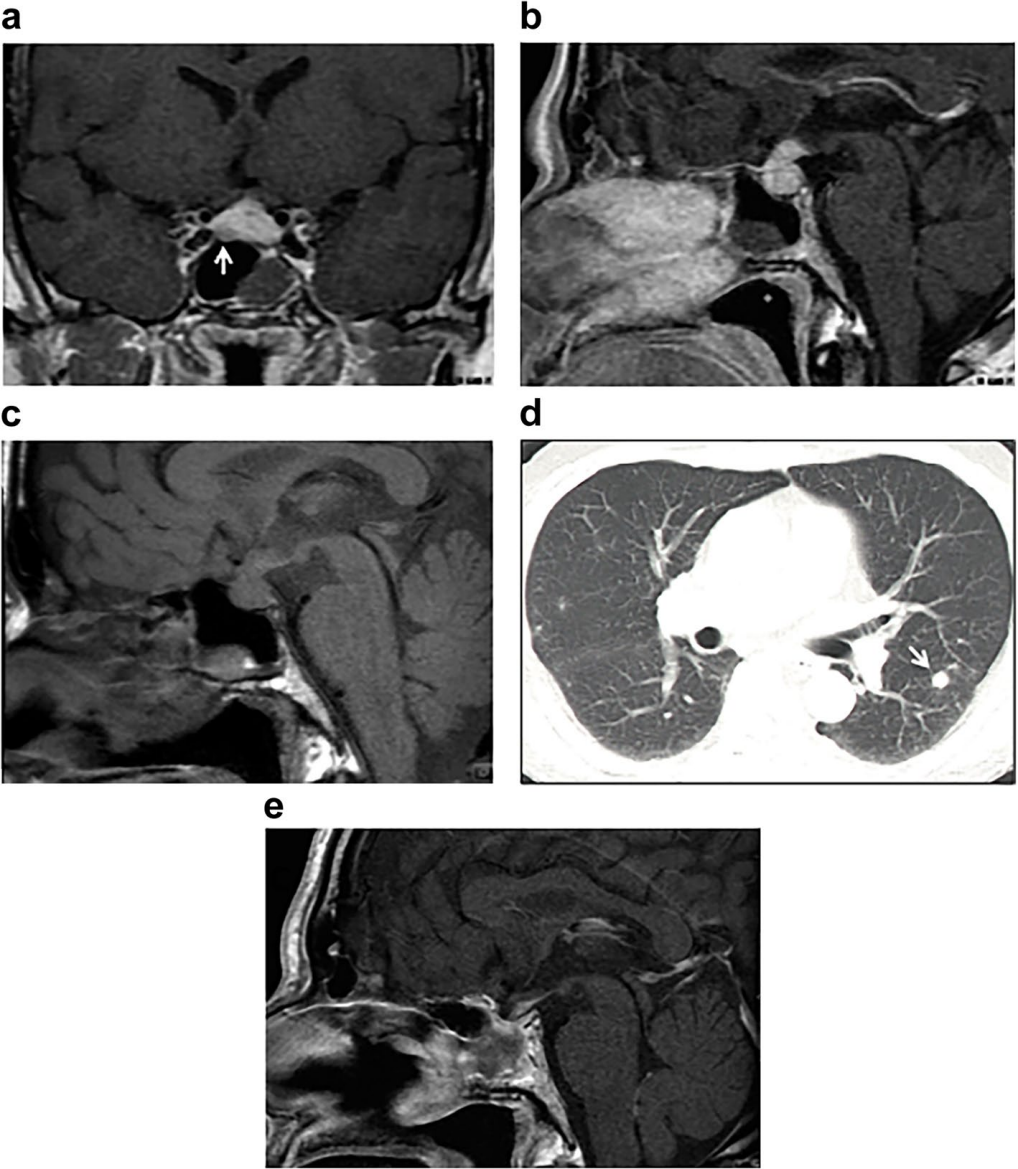
Magnetic resonance imaging (MRI) showing swelling of the pituitary body and stalk (**a**) and a 7-mm poorly enhanced mass in the right anterior lobe (**b**: arrow) before biopsy. On non-contrast MRI T1-weighted images, the high signal in the posterior pituitary lobes had disappeared (**c**). Chest computed tomography showed a nodular lesion in the left lung field (**d**: arrow). On MRI, the pituitary gland was found to be reduced in size after prednisolone treatment for 2 months (**e**)

**Fig. 2:**
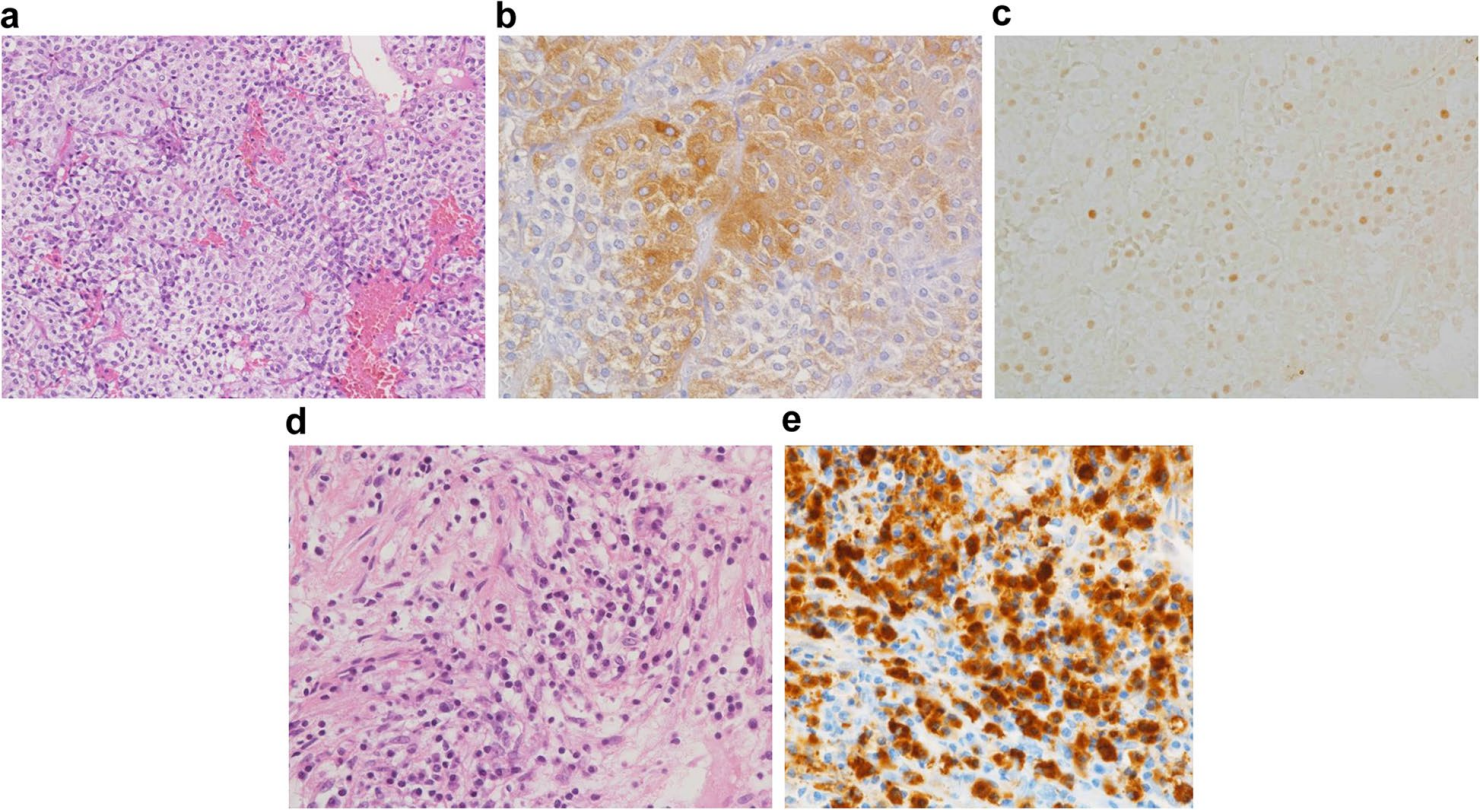
The histological features of the resected tumor are consistent with those of chromophobe pituitary neuroendocrine tumor, and there is no plasma cell infiltration (**a**: hematoxylin–eosin staining, original magnification, 400 ×). Immunohistochemistry findings indicate that the tumor is positive for follicle-stimulating hormone-beta (**b**: original magnification, 400 ×). Immunohistochemistry findings indicate the tumor is positive for steroidogenic factor-1 in the cell nuclei (**c**: original magnification, 400 ×). Pathological examination results showing a marked infiltration of plasma cells into the anterior and posterior lobes (**d**: hematoxylin–eosin staining, original magnification, 400 ×), which is more than 10 IgG4-positive plasma cells per high-power field (**e**: immunohistochemistry for IgG4 monoclonal antibody, original magnification, 400 ×)

The patient was a 56-year-old man with bitemporal hemianopia and left labial numbness. Magnetic resonance imaging (MRI) performed in another hospital showed a tumorous lesion in the pituitary aspect, and the patient was referred to our hospital. On the first hospital visit, the patient had visual impairment, bitemporal hemianopia, and right abducens nerve palsy. The results of the laboratory tests were as follows: thyroid-stimulating hormone (TSH), 0.658 μU/mL; free thyroxine, 0.41 ng/dL; growth hormone (GH), 0.62 ng/mL; luteinizing hormone, < 0.2 mIU/mL; follicle-stimulating hormone (FSH), < 1.0 mIU/mL; prolactin, 19.7 ng/mL; cortisol, 0.5 μg/dL; adrenocorticotropic hormone, 16.4 pg/mL; testosterone, < 5.0 ng/dL; and insulin-like growth factor-1, 119 ng/mL (− 0.73 standard deviation score). These findings suggested hypopituitarism. Corticotrophin-releasing hormone, GH-releasing hormone, gonadotropin-releasing hormone, and TSH loading tests showed gonadotropin, TSH, and hypothalamic–pituitary–adrenal axis deficiency (Table 1). Moreover, the GH-releasing peptide-2 loading test showed evidence of severe GH deficiency (Table 2). The patient's IgG4 level was high at 319 mg/dL (standard value, 4.8–105 mg/dL), while his IgG level was in the normal range at 1284 mg/dL. The IgG4/IgG ratio was 0.25. MRI revealed swelling of the pituitary body and stalk and a 7-mm poorly enhanced right anterior lobe mass (Fig. 1a and b). On non-contrast MRI T1-weighted images, the high signal in the posterior pituitary lobes had disappeared (Fig. 1c). The mucosa of the sphenoid sinus was thickened. A systemic examination was conducted to find other sites of IgG4 disease, and a chest computed tomography showed a nodular lesion in the left lung field (Fig. 1d). These lesions were suspected an IgG4-related disease. Other imaging studies also revealed no evidence of IgG4-related disease. The patient underwent endoscopic transnasal surgery of the pituitary gland to make a definite diagnosis. Biopsy samples were obtained from the dura mater and the anterior and posterior lobes of the pituitary gland, and the tumorous lesion in the right anterior lobe was completely resected. No other abnormal findings, such as neoplastic lesions, were observed in the pituitary region. The histological features of the resected tumor were consistent with those of chromophobe PitNET, which is positive for FSH-β and steroidogenic factor-1, with no evidence of plasma cell infiltration, and negative for IgG4 (Fig. 2a, b, and c). Pathological examination revealed a marked diffuse infiltration of plasma cells into the anterior pituitary lobe and dura, which were strongly positive for IgG4. Moreover, histopathological examination revealed marked diffuse infiltration of plasma cells into the posterior pituitary lobe (Fig. 2d), which were strongly positive for IgG4 (Fig. 2e). Based on these pathological findings, we diagnosed concurrent IgG4-related hypophysitis and gonadotroph PitNET. In addition, the diagnostic criteria for IgG4-related hypophysitis were consistent with those reported by Leporati et al. and the Japan Endocrine Society [3, 4]. This diagnosis was also reinforced by the markedly high level of IgG4 (three times higher than the upper limit of the normal level) and the suspected pulmonary involvement.

**Table 1:** Responses of pituitary and adrenal hormones to intravenous injection of corticotrophin-releasing hormone (100 µg), luteinizing hormone-releasing hormone (100 µg), and thyrotropin-releasing hormone (500 µg)

	0 min	30 min	60 min
ACTH (pg/mL)	16.0	250.0	127.5
Cortisol (µg/dL)	0.3	3.3	5.2
LH (mIU/mL)	< 1.0	1.6	2.3
FSH (mIU/mL)	< 0.2	0.9	1.2
PRL (ng/mL)	19.2	29.8	25.0
TSH (µU/mL)	0.761	4.250	2.560

*Abbreviations*: *PRL* prolactin, *TSH* thyroid-stimulating hormone, *FSH* follicle-stimulating hormone, *LH* luteinizing hormone, *ACTH* adrenocorticotropic hormone

**Table 2:** Growth hormone response to the growth hormone-releasing peptide-2 (100 µg) loading test

	0 min	15 min	30 min	45 min	60 min
GH (ng/mL)	1.3	8.2	5.9	2.1	1.4

*Abbreviation*: *GH* growth hormone

Postoperatively, diabetes insipidus developed after the administration of a steroid (20 mg/day hydrocortisone). This was a finding that suggested masked diabetes insipidus. On MRI, the pituitary gland was found to be reduced in size (Fig. 1e). Based on histopathological findings, hydrocortisone was changed to prednisolone (0.6 mg/kg/day), which further reduced the size of the pituitary gland and pulmonary lesions. Simultaneously, serum IgG and IgG4 levels returned to 1035 mg/dL and 124 mg/dL, respectively.

## Discussion and Conclusions

In 2011, Leporati proposed five criteria for diagnosing IgG4-related hypophysitis [3]. Although more than 30 cases of IgG4-related hypophysitis have been reported, only a few patients have undergone biopsy [5–7]. Furthermore, no previous report has pathologically demonstrated the coexistence of PitNET and IgG4-related hypophysitis. However, IgG4-related hypophysitis requires a careful differential diagnosis from other pituitary diseases such as PitNET, Rathke's cleft cysts, craniopharyngiomas, malignant lymphomas, and granulomatosis with polyangiitis, which sometimes accompany the secondary infiltration with small amounts of IgG4-positive plasma cells [4]. However, in this case, the possibility of an inflammatory spillover of such lesions into the normal pituitary gland is low. This observation was made based on the fact that PitNET, which was in contact with the anterior pituitary in this case, showed no evidence of inflammation or pituitary apoplexy. In addition, the patient had a pulmonary lesion other than the pituitary gland lesion, which was reduced by administering a pharmacological dose of steroids. Nishioka et al. reported that clusters of IgG4-positive plasma cells can be observed in the cases of secondary inflammation caused by other lesions [8], which is inconsistent with diffuse and strong IgG4 positivity was seen in this case. Therefore, this case is not consistent with the diffuse strong positivity for IgG4. Therefore, this case is a coexistent case of PitNET and IgG4-related hypophysitis.

Whether the occurrence of PitNET is associated with IgG4-related hypophysitis remains unclear. Recently, many studies have been conducted on the relationship between IgG4-related disease and malignant neoplasms. IgG4-related disease is diagnosed using imaging, blood tests, and pathological examinations. However, patients with pancreatic cancer have elevated serum IgG4 levels and IgG4-positive plasma cell infiltration in tumor tissue [9]. Moreover, several studies have described cases of IgG4-related disease complicated by malignant neoplasms, such as cases of pancreatic cancer occurring during the course of autoimmune pancreatitis [10] and cases of malignant lymphoma occurring during the course of Mikulicz disease [11], suggesting a relationship between the two. In their study of 114 patients with IgG4-related disease, Zen et al. discovered that 4 (3.5%) had a history of malignant neoplasm and 3 (2.6%) had malignant neoplasms after being diagnosed with IgG4-related disease [12]. Another study found that during a mean observation period of 3.1 years, 10.4% of 106 patients with IgG4-related disease had malignant neoplasms, which was 3.5 times the rate observed in healthy individuals [13].

Several mechanisms have been proposed to explain the relationship between IgG4-related disease and malignant neoplasms. Regulatory T cells (Tregs) are significantly elevated in patients with IgG4-related disease [14], and Th2 cytokines are dominant [15]. Tregs are significantly elevated in the peripheral blood and tumor tissue of patients with stomach and esophageal cancers [16], suggesting that IgG4-related disease and similar immune disorders are related to the immuno-escape mechanism of malignant tumors. Furthermore, T lymphocytes in the peripheral blood of patients with digestive cancer are Th2-dominant [17] and patients with stomach cancer have high levels of the Th2 cytokine interleukin-10 [18]. Therefore, we wondered whether there was a relationship between PitNET and IgG4-related hypophysitis in the patient in this case report. Secondary hypophysitis in the normal surrounding pituitary tissue is caused by pituitary apoplexy. To resolve our doubts, we conducted a detailed pathological study; however, the findings revealed no inflammatory cell infiltration into the pituitary tumor related to IgG4-related hypophysitis. We concluded that there was little correlation because we found no IgG4-positive cells.

Furthermore, tumor lesions often occur incidentally in the pituitary gland. Some types of incidental lesions were found in 17.8% of 1,000 cases of pituitary gland autopsy, and when restricted to lesions of ≥ 2 mm (the minimum size that can be identified on MRI), the incidence rate is 6.1%. Of those, PitNET accounted for 2% and Rathke’s cleft cyst for 3.7%, indicating that when treating pituitary lesions [19], these types of incidental lesions must be considered. Double PitNETs may also be present in the pituitary gland and occur in 0.9% of autopsy cases [20] and in 0.367%–2.6% of cases of surgery and case reports [21–23]. The patient in this case report also presented with multiple lesions, but because different levels of MRI contrast enhancement produced different results, differentiating between the lesions was difficult. Because of the major differences in treatment strategies for the two conditions, detailed imaging studies are required in patients with PitNET complicated by hypophysitis, such as our patient.

Herein, we described the case of a patient with IgG4-related hypophysitis complicated and continuous with gonadotroph PitNET. Although no pathological findings indicated a relationship between the two conditions, we were able to preoperatively differentiate multiple lesions using detailed diagnostic imaging. Incidental lesions in the pituitary gland are occasionally observed, and because multiple lesions are possible, detailed preoperative diagnosis is required when treating pituitary lesions.

## Data Availability

Due to the nature of this research, participants of this study did not agree for their data to be shared publicly, so supporting data is not available.

## Abbreviations

IgG4: Immunoglobulin G4
PitNET: Pituitary neuroendocrine tumor
FSH: Follicle-stimulating hormone
MRI: Magnetic resonance imaging
TSH: Thyroid-stimulating hormone
GH: Growth hormone
Tregs: Regulatory T cells
